# Bis(2-meth­oxy­phen­yl)(phen­yl)phosphine selenide

**DOI:** 10.1107/S1600536810051317

**Published:** 2010-12-11

**Authors:** Alfred Muller

**Affiliations:** a Research Centre in Synthesis and Catalysis, Department of Chemistry, University of Johannesburg (APK Campus), PO Box 524, Auckland Park, Johannesburg 2006, South Africa

## Abstract

The title compound, C_20_H_19_O_2_PSe or SePPh(2-OMe-C_6_H_3_)_2_, crystallizes with two distinct orientations for the meth­oxy groups. The Se=P bond is 2.1170 (7) Å and the cone angle is 176.0°. Intra­molecular C—H⋯Se inter­actions occur. In the crystal, mol­ecules are linked by inter­molecular C—H⋯Se inter­actions.

## Related literature

For bond-length data, see: Allen *et al.* (2002 ▶). For our study of phospho­rus ligands, see: Muller *et al.* (2006 ▶, 2008 ▶). For the cone angle, see: Tolman (1977 ▶). For the synthesis of *ortho-*substituted aryl­alkyl­phosphanes, see: Riihimäki *et al.* (2003 ▶).
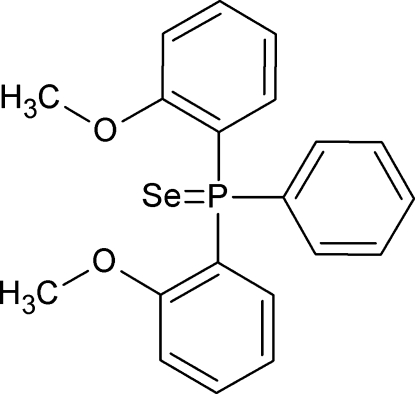


## Experimental

### 

#### Crystal data

C_20_H_19_O_2_PSe
*M* *_r_* = 401.28
Monoclinic, 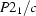

*a* = 8.9552 (4) Å
*b* = 13.2737 (6) Å
*c* = 15.9593 (6) Å
β = 104.885 (1)°
*V* = 1833.40 (14) Å^3^
*Z* = 4
Mo *K*α radiation
μ = 2.14 mm^−1^
*T* = 100 K
0.1 × 0.07 × 0.06 mm

#### Data collection

Bruker X8 APEXII 4K Kappa CCD diffractometer
Absorption correction: multi-scan (*SADABS*; Bruker, 2004 ▶) *T*_min_ = 0.814, *T*_max_ = 0.882
37384 measured reflections
5078 independent reflections
3483 reflections with *I* > 2σ(*I*)
*R*_int_ = 0.068

#### Refinement

*R*[*F* ^2^ > 2σ(*F* ^2^)] = 0.039
*wR*(*F* ^2^) = 0.096
*S* = 1.05
5078 reflections
219 parameters
H-atom parameters constrained
Δρ_max_ = 0.66 e Å^−3^
Δρ_min_ = −0.56 e Å^−3^

### 

Data collection: *APEX2* (Bruker, 2005 ▶); cell refinement: *SAINT-Plus* (Bruker, 2004 ▶); data reduction: *SAINT-Plus* and *XPREP* (Bruker, 2004 ▶); program(s) used to solve structure: *SIR97* (Altomare *et al.*, 1999 ▶); program(s) used to refine structure: *SHELXL97* (Sheldrick, 2008 ▶); molecular graphics: *DIAMOND* (Brandenburg & Putz, 2005 ▶); software used to prepare material for publication: *WinGX* (Farrugia, 1999 ▶).

## Supplementary Material

Crystal structure: contains datablocks global, I. DOI: 10.1107/S1600536810051317/kp2295sup1.cif

Structure factors: contains datablocks I. DOI: 10.1107/S1600536810051317/kp2295Isup2.hkl

Additional supplementary materials:  crystallographic information; 3D view; checkCIF report

## Figures and Tables

**Table 1:** Hydrogen-bond geometry (Å, °)

*D*—H⋯*A*	*D*—H	H⋯*A*	*D*⋯*A*	*D*—H⋯*A*
C13—H13⋯Se1^i^	0.95	2.9	3.775 (3)	154
C15—H15⋯Se1^ii^	0.95	2.96	3.740 (3)	140
C16—H16⋯Se1	0.95	2.75	3.333 (3)	120
C32—H32⋯Se1	0.95	2.97	3.462 (3)	114

Symmetry codes: (i) 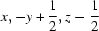; (ii) 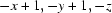.

**Fig. 1.**
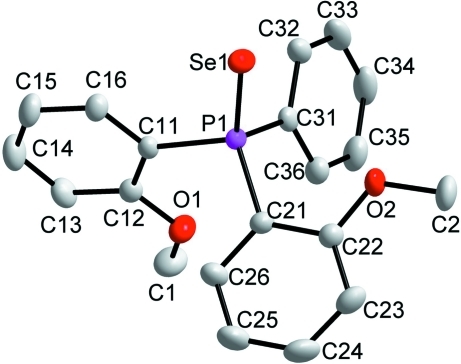
View of (I) (50% probability displacement ellipsoids). H atoms have been omitted for clarity. For the C atoms, the first digit indicates the ring number and the second digit indicates the position of the atom in the ring.

## supplementary crystallographic information

### Comment

The evaluation of the electronic parameter of tertiary phosphines is a study that spans over several decades, and even today attracts attention due to its importance. As part of a systematic investigation we are studying selenium bonded phosphorus ligands (see Muller *et al.* 2008) to give insight on the nature of these ligands. There is no steric crowding effect, albeit crystal packing effects, as normally found in transition metal complexes with bulky ligands, *e.g.* in *trans*-[Rh(CO)Cl{P(OC_6_H_5_)_3_}_2_] cone angle variations from 156° to 167° was observed for the two phosphite ligands (Muller, *et al.* 2006). Using the geometries obtained from the selenium bonded phosphorus ligands and the ^31^P NMR *J*(^31^P-^77^Se) couplings, it would be possible to obtain more information regarding the nature of the phosphorous substituted ligands.

Geometrical parameters of the molecule are as expected (Allen, 2002). Selenium atom and the three aryl groups adopt a distorted tetrahedral arrangement about phosphorous (Fig. 1). The cone angle of 176.0 ° can be calculated for the Se—P distance adjusted to 2.28 Å (the default value from Tolman, 1977). The cone value observed in the title compound is close to the value 178 (7) ° calculated from data of 5 metal bonded phosphines extracted from Cambridge Structural Database (Version 5.31, update of August; Allen, 2002).

Two different orientations for the methoxy moieties might be explained by some weak interactions (Table 2) forcing them into the conformations observed.

### Experimental

PPh(2-OMe-C_6_H_4_)_2_ were prepared either by direct ortho metallation of anisole with BuLi followed the addition of the appropriate chlorophosphine or by metal/halogen exchange between BuLi and 1-bromo-2-methoxybenzene  followed by the addition of PPhCl_2_ according to established methods (Riihimäki *et al.* 2003).

Eqimolar amounts of KSeCN and the PPh(2-OMe-C_6_H_4_)_2_ compound (ca. 0.04 mmol) were dissolved in the minimum amounts of methanol (10 – 20 mL). The KSeCN solution was added drop wise (5 min.) to the phosphine solution with stirring at room temperature. The final solution was left to evaporate slowly until dry to give crystals suitable for a single crystal X-ray study.

Analytical data: ^31^P {H} NMR (CDCl_3_, 121.42 MHz): For PPh(2-OMe-C_6_H_4_)_2_δ = -26.41 (s) For SePPh(2-OMe-C_6_H_4_)_2_δ = 28.42 (t, ^1^J_P-Se_ = 717.5 Hz)

### Refinement

The aromatic and methylene H atoms were placed in geometrically idealised positions (C—H = 0.93 – 0.98 Å) and constrained to ride on their parent atoms with *U*_iso_(H) = 1.2*U*_eq_(C) and *U*_iso_(H) = 1.5*U*_eq_(C) respectively, with torsion angles refined from the electron density for the methyl groups. The highest residual electron density was located 1.01 Å from Se.

### Crystal data

**Table:**

C_20_H_19_O_2_PSe	*F*(000) = 816
*M**_r_* = 401.28	*D*_x_ = 1.454 Mg m^−^^3^
Monoclinic, *P*2_1_/*c*	Mo *K*α radiation, λ = 0.71073 Å
Hall symbol: -P 2ybc	Cell parameters from 7311 reflections
*a* = 8.9552 (4) Å	θ = 2.4–28.7°
*b* = 13.2737 (6) Å	µ = 2.14 mm^−^^1^
*c* = 15.9593 (6) Å	*T* = 100 K
β = 104.885 (1)°	Cuboid, colourless
*V* = 1833.40 (14) Å^3^	0.1 × 0.07 × 0.06 mm
*Z* = 4	

### Data collection

**Table:**

Bruker X8 APEXII 4K Kappa CCD diffractometer	5078 independent reflections
graphite	3483 reflections with *I* > 2σ(*I*)
Detector resolution: 8.4 pixels mm^-1^	*R*_int_ = 0.068
ω and φ scans	θ_max_ = 29.5°, θ_min_ = 2.4°
Absorption correction: multi-scan (*SADABS*; Bruker, 2004)	*h* = −12→12
*T*_min_ = 0.814, *T*_max_ = 0.882	*k* = −18→18
37384 measured reflections	*l* = −21→21

### Refinement

**Table:**

Refinement on *F*^2^	Primary atom site location: structure-invariant direct methods
Least-squares matrix: full	Secondary atom site location: difference Fourier map
*R*[*F*^2^ > 2σ(*F*^2^)] = 0.039	Hydrogen site location: inferred from neighbouring sites
*wR*(*F*^2^) = 0.096	H-atom parameters constrained
*S* = 1.05	*w* = 1/[σ^2^(*F*_o_^2^) + (0.0352*P*)^2^ + 1.667*P*] where *P* = (*F*_o_^2^ + 2*F*_c_^2^)/3
5078 reflections	(Δ/σ)_max_ = 0.001
219 parameters	Δρ_max_ = 0.66 e Å^−^^3^
0 restraints	Δρ_min_ = −0.56 e Å^−^^3^

### Special details

**Table:**

Experimental. The intensity data was collected on a Bruker X8 Apex II 4 K Kappa CCD diffractometer using an exposure time of 20 s/frame. A total of 1897 frames were collected with a frame width of 0.5° covering up to θ = 29.48° with 99.6% completeness accomplished.
Geometry. All e.s.d.'s (except the e.s.d. in the dihedral angle between two l.s. planes) are estimated using the full covariance matrix. The cell e.s.d.'s are taken into account individually in the estimation of e.s.d.'s in distances, angles and torsion angles; correlations between e.s.d.'s in cell parameters are only used when they are defined by crystal symmetry. An approximate (isotropic) treatment of cell e.s.d.'s is used for estimating e.s.d.'s involving l.s. planes.
Refinement. Refinement of *F*^2^ against ALL reflections. The weighted *R*-factor *wR* and goodness of fit *S* are based on *F*^2^, conventional *R*-factors *R* are based on *F*, with *F* set to zero for negative *F*^2^. The threshold expression of *F*^2^ > σ(*F*^2^) is used only for calculating *R*-factors(gt) *etc*. and is not relevant to the choice of reflections for refinement. *R*-factors based on *F*^2^ are statistically about twice as large as those based on *F*, and *R*- factors based on ALL data will be even larger.

### Fractional atomic coordinates and isotropic or equivalent isotropic displacement parameters (Å^2^)

**Table:**

	*x*	*y*	*z*	*U*_iso_*/*U*_eq_	
P1	0.79534 (8)	0.29050 (5)	0.06864 (4)	0.02021 (14)	
Se1	0.73477 (4)	0.42323 (2)	0.125712 (17)	0.02827 (9)	
C11	0.6822 (3)	0.27995 (19)	−0.04376 (16)	0.0203 (5)	
C12	0.7089 (3)	0.2045 (2)	−0.09973 (16)	0.0233 (5)	
C13	0.6251 (3)	0.2023 (2)	−0.18633 (17)	0.0308 (6)	
H13	0.6437	0.1511	−0.224	0.037*	
C14	0.5148 (4)	0.2752 (3)	−0.21685 (19)	0.0377 (7)	
H14	0.4577	0.2739	−0.2759	0.045*	
C15	0.4862 (3)	0.3496 (2)	−0.16343 (18)	0.0338 (7)	
H15	0.4095	0.399	−0.1854	0.041*	
C16	0.5697 (3)	0.3522 (2)	−0.07726 (16)	0.0248 (6)	
H16	0.5499	0.4041	−0.0405	0.03*	
O1	0.8187 (2)	0.13463 (15)	−0.06510 (12)	0.0325 (5)	
C1	0.8732 (4)	0.0704 (2)	−0.1232 (2)	0.0415 (8)	
H1A	0.7933	0.0209	−0.1487	0.062*	
H1B	0.9668	0.0353	−0.0913	0.062*	
H1C	0.8967	0.1112	−0.1694	0.062*	
C21	0.7630 (3)	0.17564 (19)	0.12269 (17)	0.0237 (6)	
C22	0.8553 (3)	0.1529 (2)	0.20616 (17)	0.0265 (6)	
C23	0.8299 (4)	0.0648 (2)	0.2478 (2)	0.0374 (7)	
H23	0.8943	0.0484	0.3033	0.045*	
C24	0.7099 (4)	0.0011 (2)	0.2076 (2)	0.0439 (8)	
H24	0.6929	−0.0592	0.2359	0.053*	
C25	0.6152 (4)	0.0241 (2)	0.1272 (2)	0.0400 (8)	
H25	0.5316	−0.0189	0.1009	0.048*	
C26	0.6430 (3)	0.1103 (2)	0.08520 (19)	0.0300 (6)	
H26	0.5787	0.1254	0.0294	0.036*	
O2	0.9673 (2)	0.22128 (15)	0.24085 (12)	0.0317 (5)	
C2	1.0661 (4)	0.2012 (3)	0.32533 (19)	0.0404 (8)	
H2A	1.0043	0.1997	0.3679	0.061*	
H2B	1.1444	0.2543	0.3409	0.061*	
H2C	1.117	0.1359	0.3249	0.061*	
C31	0.9970 (3)	0.2904 (2)	0.06549 (16)	0.0235 (5)	
C32	1.0691 (3)	0.3820 (2)	0.05993 (17)	0.0307 (6)	
H32	1.0143	0.4433	0.0597	0.037*	
C33	1.2217 (4)	0.3836 (3)	0.0548 (2)	0.0405 (8)	
H33	1.2704	0.4461	0.0499	0.049*	
C34	1.3027 (4)	0.2952 (3)	0.05663 (19)	0.0413 (8)	
H34	1.407	0.2968	0.0532	0.05*	
C35	1.2326 (4)	0.2042 (3)	0.0634 (2)	0.0387 (7)	
H35	1.2887	0.1432	0.0648	0.046*	
C36	1.0799 (3)	0.2014 (2)	0.06837 (18)	0.0302 (6)	
H36	1.0321	0.1386	0.0737	0.036*	

### Atomic displacement parameters (Å^2^)

**Table:**

	*U*^11^	*U*^22^	*U*^33^	*U*^12^	*U*^13^	*U*^23^
P1	0.0238 (3)	0.0198 (3)	0.0160 (3)	−0.0016 (3)	0.0031 (3)	0.0015 (2)
Se1	0.04196 (18)	0.02334 (14)	0.02004 (13)	0.00041 (13)	0.00894 (11)	−0.00166 (11)
C11	0.0204 (13)	0.0229 (12)	0.0172 (12)	−0.0029 (10)	0.0042 (10)	0.0016 (10)
C12	0.0245 (14)	0.0251 (13)	0.0207 (13)	−0.0018 (11)	0.0066 (11)	0.0018 (10)
C13	0.0346 (16)	0.0380 (16)	0.0201 (13)	−0.0036 (13)	0.0075 (12)	−0.0070 (12)
C14	0.0349 (17)	0.055 (2)	0.0203 (14)	0.0018 (15)	0.0012 (12)	−0.0016 (14)
C15	0.0277 (15)	0.0454 (18)	0.0257 (14)	0.0090 (13)	0.0021 (12)	0.0057 (13)
C16	0.0237 (14)	0.0315 (14)	0.0197 (12)	0.0023 (11)	0.0066 (10)	0.0019 (11)
O1	0.0420 (12)	0.0289 (11)	0.0252 (10)	0.0084 (9)	0.0059 (9)	−0.0014 (8)
C1	0.054 (2)	0.0318 (16)	0.0398 (18)	0.0110 (15)	0.0138 (15)	−0.0044 (14)
C21	0.0283 (15)	0.0211 (12)	0.0219 (13)	−0.0011 (10)	0.0068 (11)	0.0023 (10)
C22	0.0294 (15)	0.0259 (14)	0.0240 (13)	−0.0015 (12)	0.0062 (11)	0.0026 (11)
C23	0.0465 (19)	0.0331 (16)	0.0324 (16)	0.0027 (14)	0.0097 (14)	0.0134 (13)
C24	0.064 (2)	0.0269 (15)	0.0427 (19)	−0.0082 (16)	0.0174 (17)	0.0090 (14)
C25	0.049 (2)	0.0314 (16)	0.0401 (18)	−0.0156 (14)	0.0119 (15)	0.0000 (13)
C26	0.0355 (17)	0.0276 (14)	0.0271 (14)	−0.0092 (12)	0.0083 (12)	−0.0004 (11)
O2	0.0335 (11)	0.0362 (11)	0.0200 (9)	−0.0051 (9)	−0.0031 (8)	0.0062 (8)
C2	0.0351 (18)	0.051 (2)	0.0277 (16)	0.0066 (15)	−0.0059 (13)	0.0060 (14)
C31	0.0256 (14)	0.0280 (14)	0.0153 (12)	−0.0040 (11)	0.0021 (10)	0.0015 (10)
C32	0.0339 (16)	0.0315 (15)	0.0238 (14)	−0.0094 (12)	0.0024 (12)	0.0050 (12)
C33	0.0368 (18)	0.052 (2)	0.0302 (16)	−0.0214 (16)	0.0039 (14)	0.0105 (14)
C34	0.0243 (15)	0.070 (2)	0.0283 (16)	−0.0084 (16)	0.0037 (12)	0.0134 (16)
C35	0.0276 (16)	0.053 (2)	0.0331 (16)	0.0055 (15)	0.0036 (13)	0.0103 (15)
C36	0.0261 (15)	0.0331 (15)	0.0293 (15)	−0.0013 (12)	0.0031 (12)	0.0044 (12)

### Geometric parameters (Å, °)

**Table:**

P1—C21	1.811 (3)	C23—C24	1.388 (5)
P1—C31	1.820 (3)	C23—H23	0.95
P1—C11	1.825 (3)	C24—C25	1.379 (5)
P1—Se1	2.1170 (7)	C24—H24	0.95
C11—C16	1.395 (4)	C25—C26	1.381 (4)
C11—C12	1.403 (4)	C25—H25	0.95
C12—O1	1.361 (3)	C26—H26	0.95
C12—C13	1.393 (4)	O2—C2	1.435 (3)
C13—C14	1.379 (4)	C2—H2A	0.98
C13—H13	0.95	C2—H2B	0.98
C14—C15	1.370 (4)	C2—H2C	0.98
C14—H14	0.95	C31—C32	1.389 (4)
C15—C16	1.387 (4)	C31—C36	1.390 (4)
C15—H15	0.95	C32—C33	1.390 (4)
C16—H16	0.95	C32—H32	0.95
O1—C1	1.435 (3)	C33—C34	1.376 (5)
C1—H1A	0.98	C33—H33	0.95
C1—H1B	0.98	C34—C35	1.378 (5)
C1—H1C	0.98	C34—H34	0.95
C21—C26	1.391 (4)	C35—C36	1.390 (4)
C21—C22	1.408 (4)	C35—H35	0.95
C22—O2	1.361 (3)	C36—H36	0.95
C22—C23	1.392 (4)		
			
C21—P1—C31	107.09 (12)	C24—C23—C22	119.6 (3)
C21—P1—C11	106.69 (12)	C24—C23—H23	120.2
C31—P1—C11	106.10 (11)	C22—C23—H23	120.2
C21—P1—Se1	113.94 (9)	C25—C24—C23	120.8 (3)
C31—P1—Se1	112.21 (9)	C25—C24—H24	119.6
C11—P1—Se1	110.36 (9)	C23—C24—H24	119.6
C16—C11—C12	118.1 (2)	C24—C25—C26	119.4 (3)
C16—C11—P1	119.21 (19)	C24—C25—H25	120.3
C12—C11—P1	122.6 (2)	C26—C25—H25	120.3
O1—C12—C13	122.5 (2)	C25—C26—C21	121.6 (3)
O1—C12—C11	116.8 (2)	C25—C26—H26	119.2
C13—C12—C11	120.7 (2)	C21—C26—H26	119.2
C14—C13—C12	119.3 (3)	C22—O2—C2	118.1 (2)
C14—C13—H13	120.3	O2—C2—H2A	109.5
C12—C13—H13	120.3	O2—C2—H2B	109.5
C15—C14—C13	121.1 (3)	H2A—C2—H2B	109.5
C15—C14—H14	119.4	O2—C2—H2C	109.5
C13—C14—H14	119.4	H2A—C2—H2C	109.5
C14—C15—C16	119.8 (3)	H2B—C2—H2C	109.5
C14—C15—H15	120.1	C32—C31—C36	119.5 (3)
C16—C15—H15	120.1	C32—C31—P1	118.9 (2)
C15—C16—C11	121.0 (3)	C36—C31—P1	121.6 (2)
C15—C16—H16	119.5	C31—C32—C33	119.9 (3)
C11—C16—H16	119.5	C31—C32—H32	120.1
C12—O1—C1	118.2 (2)	C33—C32—H32	120.1
O1—C1—H1A	109.5	C34—C33—C32	120.4 (3)
O1—C1—H1B	109.5	C34—C33—H33	119.8
H1A—C1—H1B	109.5	C32—C33—H33	119.8
O1—C1—H1C	109.5	C33—C34—C35	120.1 (3)
H1A—C1—H1C	109.5	C33—C34—H34	120
H1B—C1—H1C	109.5	C35—C34—H34	120
C26—C21—C22	118.3 (2)	C34—C35—C36	120.2 (3)
C26—C21—P1	121.3 (2)	C34—C35—H35	119.9
C22—C21—P1	120.3 (2)	C36—C35—H35	119.9
O2—C22—C23	124.1 (2)	C35—C36—C31	120.0 (3)
O2—C22—C21	115.7 (2)	C35—C36—H36	120
C23—C22—C21	120.2 (3)	C31—C36—H36	120
			
C21—P1—C11—C16	121.2 (2)	P1—C21—C22—O2	−0.7 (3)
C31—P1—C11—C16	−124.8 (2)	C26—C21—C22—C23	2.7 (4)
Se1—P1—C11—C16	−3.0 (2)	P1—C21—C22—C23	179.7 (2)
C21—P1—C11—C12	−62.0 (2)	O2—C22—C23—C24	178.4 (3)
C31—P1—C11—C12	51.9 (2)	C21—C22—C23—C24	−2.1 (5)
Se1—P1—C11—C12	173.69 (19)	C22—C23—C24—C25	−0.2 (5)
C16—C11—C12—O1	−179.6 (2)	C23—C24—C25—C26	1.8 (5)
P1—C11—C12—O1	3.6 (3)	C24—C25—C26—C21	−1.1 (5)
C16—C11—C12—C13	0.1 (4)	C22—C21—C26—C25	−1.2 (4)
P1—C11—C12—C13	−176.6 (2)	P1—C21—C26—C25	−178.1 (2)
O1—C12—C13—C14	179.6 (3)	C23—C22—O2—C2	0.3 (4)
C11—C12—C13—C14	−0.1 (4)	C21—C22—O2—C2	−179.2 (2)
C12—C13—C14—C15	−0.1 (5)	C21—P1—C31—C32	−154.9 (2)
C13—C14—C15—C16	0.3 (5)	C11—P1—C31—C32	91.4 (2)
C14—C15—C16—C11	−0.3 (4)	Se1—P1—C31—C32	−29.2 (2)
C12—C11—C16—C15	0.1 (4)	C21—P1—C31—C36	25.2 (2)
P1—C11—C16—C15	177.0 (2)	C11—P1—C31—C36	−88.5 (2)
C13—C12—O1—C1	13.8 (4)	Se1—P1—C31—C36	150.9 (2)
C11—C12—O1—C1	−166.5 (2)	C36—C31—C32—C33	1.9 (4)
C31—P1—C21—C26	−127.6 (2)	P1—C31—C32—C33	−178.0 (2)
C11—P1—C21—C26	−14.3 (3)	C31—C32—C33—C34	−1.1 (4)
Se1—P1—C21—C26	107.7 (2)	C32—C33—C34—C35	0.2 (5)
C31—P1—C21—C22	55.5 (2)	C33—C34—C35—C36	0.1 (5)
C11—P1—C21—C22	168.8 (2)	C34—C35—C36—C31	0.7 (4)
Se1—P1—C21—C22	−69.2 (2)	C32—C31—C36—C35	−1.6 (4)
C26—C21—C22—O2	−177.7 (2)	P1—C31—C36—C35	178.2 (2)

### Hydrogen-bond geometry (Å, °)

**Table:**

*D*—H···*A*	*D*—H	H···*A*	*D*···*A*	*D*—H···*A*
C13—H13···Se1^i^	0.95	2.9	3.775 (3)	154
C15—H15···Se1^ii^	0.95	2.96	3.740 (3)	140
C16—H16···Se1	0.95	2.75	3.333 (3)	120
C32—H32···Se1	0.95	2.97	3.462 (3)	114

Symmetry codes: (i) *x*, −*y*+1/2, *z*−1/2; (ii) −*x*+1, −*y*+1, −*z*.
